# Nace una revista para avanzar en la Medicina de Laboratorio

**DOI:** 10.1515/almed-2019-0007

**Published:** 2019-12-24

**Authors:** Imma Caballé

**Affiliations:** Presidenta Sociedad Española de Medicina de Laboratorio, Barcelona, Spain

La difusión del conocimiento científico requiere disponer de medios suficientes que permitan conseguir exitosamente tal objetivo. Publicar es fundamental en la investigación científica porque permite el acceso a hallazgos importantes para la comunidad científica y la sociedad.

Asimismo, la publicación de artículos forma parte del currículo profesional ya que proporciona un medio objetivo para la evaluación de progreso académico, permite solicitar fondos para proyectos y la evaluación comparativa de científicos individuales, universidades y otras instituciones científicas.

Disponer de una nueva revista científica para la Sociedad Española de Medicina de Laboratorio representa un factor clave para la contribuir al conocimiento científico y al desarrollo profesional. Así pues, por considerarlo de una importancia capital para todos nuestros profesionales, se incluyó como un objetivo de la SEQC^ML^ en el Plan Estratégico 2018–2020.

Además, los socios y en especial los componentes del Comité Científico llevan años reclamando una publicación indexada y la posibilidad de publicar en inglés. Por ello, la Junta Directiva de la SEQC^ML^ ha decidido embarcarse en un ambicioso proyecto de importancia clave para la difusión e intercambio de información científica.

A partir del 2020, nuestra Sociedad tendrá una revista propia, online, Open Access y bilingüe (español/inglés) que pretende lograr una mayor difusión de la literatura científica relacionada con nuestra especialidad.

La nueva revista pretende facilitar la divulgación de artículos originales y documentos científicos relacionados con la Medicina de Laboratorio en una publicación de alto impacto y amplia difusión.

Entre los posibles criterios relacionados con la evaluación de la actividad científica ejerce un papel destacado el número de artículos publicados por el científico (a lo largo de toda la carrera científica o dentro de un período de tiempo determinado) junto con el impacto de la investigación publicada, tanto en términos de factor de impacto de la revista donde se publican los artículos y la cantidad de citas de estos artículos. El uso de estos “índices bibliométricos” ha contribuido enormemente impulsar la publicación científica en los últimos años.

Si bien cabe señalar que el factor de impacto como indicador ha originado controversias que se mantienen abiertas [1], [2], también es cierto que han aparecido alternativas y ha habido progresos para mejorar su diseño y utilización [3]. Además del factor de impacto del Journal of Citation Reports, hay el SCImago y Google Scholar. En cualquier caso, la nueva revista nace con el objetivo de ocupar una presencia significativa en los índices de factores de impacto ya que será un reflejo del resultado obtenido. En este sentido se ha configurado un equipo editorial de gran cualificación y experiencia que contribuirá decisivamente a conseguirlo y desde aquí invitamos a la comunidad científica para que aporten sus artículos.

En la actualidad la lengua fundamental de comunicación científica es el inglés. Pero al mismo tiempo consideramos necesario que el acceso a la información pueda llegar a ámbitos donde el inglés pueda ser una barrera. Por este motivo se ha considerado oportuno que la publicación sea bilingüe, en inglés y en español. Esto facilitará tanto a investigadores y profesionales del laboratorio que su actividad científica tenga repercusión.

La evolución de las plataformas de publicación científica en la difusión de contenido digital nos ha conducido a considerar que la nueva revista sea de acceso abierto. Entendemos que facilitar el acceso es una decisión que contribuirá en positivo para alcanzar un mayor factor de impacto que es precisamente lo que se pretende.

La revista aparece con el nombre de Avances en Medicina de Laboratorio y precisamente bajo este título aspiramos a que la innovación fruto de la aplicación del conocimiento científico tenga una traducción práctica en la Medicina de Laboratorio.

El valor último del laboratorio clínico se encuentra en la información que permite detectar o cambiar el curso de la enfermedad y con ello contribuir a la salud poblacional. Este es el fin último de la revista que hoy nace y al que se compromete firmemente.

Estamos ante un proyecto ilusionante para todos y es fundamental la implicación y colaboración de todos para conseguir una revista de calidad, un medio de difusión del conocimiento científico en la Medicina de Laboratorio.
